# Intraglandular dissemination: a special pathological feature

**DOI:** 10.3389/fonc.2024.1428274

**Published:** 2024-07-29

**Authors:** Yubi Zhang, Yu Deng, Meng Zhou, Bin Wu, Jing Zhou

**Affiliations:** ^1^ Department of Thyroid and Breast Surgery, Union Hospital, Tongji Medical College, Huazhong University of Science and Technology, Wuhan, China; ^2^ Department of Orthopedics, Union Hospital, Tongji Medical College, Huazhong University of Science and Technology, Wuhan, China; ^3^ Department of Thyroid and Breast Surgery, People’s Hospital of Dongxihu District Wuhan City and Union Dongxihu Hospital, Huazhong University of Science and Technology, Wuhan, China

**Keywords:** thyroid carcinoma (TC), papillary thyroid carcinoma (PTC), intraglandular dissemination, lymph node metastasis, BRAF V600E mutation

## Abstract

Intraglandular dissemination is an important pathological feature of thyroid cancer, yet the biological characteristics of this phenomenon remain relatively underexplored. This paper aims to provide a comprehensive overview of its biological behaviors, protein expressions, and identification methods. Several retrospective studies have found that thyroid cancers with intraglandular dissemination have higher rates of lymph node metastasis, capsule invasion, and vascular invasion, exhibiting more aggressive biological behavior. Immunohistochemistry results show abnormal expression of proteins such as FKBP5, CENPF, CX26, KIF11, PTK7, which are associated with poor prognosis in thyroid cancers with intraglandular dissemination, offering potential guidance for specific targeted therapy in the future. Moreover, adjunctive techniques including ultrasound, fine-needle aspiration, and genetic testing offer valuable support in accurately identifying these cases, facilitating moreproactive treatment and closer follow-up.

## 1 Introduction

Thyroid cancer is the most common endocrine tumor, with its incidence steadily increasing year by year. It has become the seventh most common tumor in China, with a particularly noticeable upward trend among females. The latest data shows that the incidence of thyroid cancer in females has risen from seventh place to third (1). Although the overall prognosis of thyroid cancer is good, with a five-year survival rate as high as 97.5% (2), approximately 35% of patients experience recurrence after initial surgical treatment (3). Studies indicate that thyroid cancers with intraglandular dissemination exhibit more aggressive biological behavior (4, 5). Patients with intraglandular dissemination have a significantly higher rate of neck lymph node recurrence under the same treatment regimen (total thyroidectomy + postoperative I131), leading to an increased risk of reoperation (4). Therefore, more attention should be paid to this type of thyroid cancer.

Currently, there is no unified definition or diagnostic criteria for intraglandular dissemination. Although no precise definition has been proposed in the literature, the characteristics of intraglandular dissemination can be summarized based on relevant cases: there is usually a larger primary lesion; around the primary lesion, there are several small scattered lesions arranged radially. The diameter of these scattered lesions is typically less than 4mm, and as the distance from the primary lesion increases, the number of small scattered lesions decreases (6, 7).

Previous studies have suggested that intraglandular dissemination of thyroid cancer results from tumor cells disseminationing through intraglandular lymphatic vessels (8, 9). However, Fernandez-Cruz L, through thyroid lymphography, discovered that each lobe of the thyroid gland has its own lymphatic system, with no lymphatic connection between the two lobes. When contrast medium is injected into one lobe of the thyroid gland, only the lymph nodes on the same side of the neck are visualized. Therefore, researchers have raised questions about the dissemination of cancer cells through lymphatic vessels from one lobe to the other in cases of bilateral involvement with intraglandular dissemination of thyroid cancer (10). Further research is needed to elucidate the specific mechanisms underlying such intraglandular dissemination.

Regarding the incidence of intraglandular dissemination, different studies have shown varying results due to differences in study populations. A study from 1963 found the probability of intraglandular dissemination of thyroid cancer to be 18% (9), while a study from 2021 indicated an occurrence rate of 13.2% (11). However, due to the relatively long time span between these studies and the continuous upward trend in thyroid cancer incidence, the recent incidence of thyroid cancer with intraglandular dissemination may have also undergone significant changes. Therefore, it is urgently necessary for the latest research to provide updated statistics on this matter.

Given that thyroid cancer with intraglandular dissemination exhibits stronger invasiveness and a higher incidence rate, this paper aims to provide an overview of the clinical characteristics, biological behavior, protein expressions, as well as preoperative and postoperative identification methods of thyroid cancer with intraglandular dissemination. This summary will facilitate the development of more accurate treatment and management strategies for such patients in the future.

## 2 The clinical features of thyroid cancer with intraglandular dissemination

Thyroid cancer with intraglandular dissemination exhibits stronger invasiveness, higher recurrence rates, and increased rates of reoperation compared to typical thyroid cancer. As papillary thyroid carcinoma (PTC) is the most common pathological type of thyroid cancer, accounting for over 80% (12), current research on intraglandular dissemination mainly focuses on PTC patients. Therefore, the clinical features of thyroid cancer with intraglandular dissemination are primarily conducted within the PTC population.

Firstly, regarding gender, previous studies have indicated that female is a risk factor for papillary thyroid carcinoma (PTC) (13). However, current research suggests that male is the risk factor for PTC with intraglandular dissemination. In 2013, Džepina D et al. analyzed the pathological and clinical characteristics of 714 PTC patients (undergoing total thyroidectomy and neck lymph node dissection). The results showed that males were more likely to exhibit intraglandular dissemination (p = 0.015) and were more prone to have dissemination on the contralateral lobe compared to females (31.5% in males vs. 19.9% in females) (14). Secondly, compared to typical PTC, a study by Lei Y et al. (comprising 25 cases of intraglandular disseminated PTC, 17 cases of multifocal non-intraglandular disseminated PTC, and 31 cases of solitary PTC) found that PTC with intraglandular dissemination had a lower overall onset age (5).

The association between tumor size and the occurrence of intraglandular dissemination has garnered significant attention. Does a larger tumor diameter heighten the likelihood of intraglandular dissemination? However, various studies have produced conflicting findings. Qian B et al. conducted a thorough analysis and validation of data from 145,951 PTC patients in the SEER database and 8,751 PTC patients from HUST Union Hospital. They discovered a correlation between intraglandular dissemination and tumor size. Compared to the PTC group, the incidence of intraglandular dissemination was lower in the PTMC group with tumors smaller than 10mm in diameter (15). Similarly, Džepina D et al. observed a significant statistical relationship between tumors larger than 10mm in diameter and intraglandular dissemination (14). However, an alternative perspective was presented by another study, suggesting the absence of a clear association between intraglandular dissemination and tumor size. Miki H et al. conducted a comparison of the pathological characteristics between 38 cases with tumors <10mm in diameter and 74 cases with tumors >10mm in diameter of papillary thyroid carcinoma. Their results indicated an intraglandular dissemination rate of 26% for tumors <10mm in diameter and 27% for tumors >10mm in diameter, with no statistical difference between the two groups (16). The opposite findings of these studies, attributed to variations in study populations, so more research is needed to elucidate the relationship between tumor size and intraglandular dissemination.

Moreover, previous research indicates that if first-degree relatives within a family are afflicted with non-medullary thyroid cancer (FNMTC: papillary carcinoma, follicular carcinoma), the risk of thyroid cancer escalates significantly for other family members (17, 18). Does a similar correlation exist between the occurrence of intraglandulardissemination and a familial history of thyroid cancer? In 2002, Uchino S et al. conducted a comparative analysis of the clinical characteristics of sporadic and familial non-medullary thyroid cancer (defined as having at least one first-degree relative with the disease). The findings revealed that in comparison to sporadic cases, FNMTC patients were more prone to intraglandular dissemination (28.5% vs. 40.7%), with a higher recurrence rate (16.3% vs. 9.6%) (19). However, research on the correlation between family history and intraglandular dissemination remains scarce to date. Whether a family history serves as a risk factor for intraglandular dissemination warrants deliberation and necessitates further evidence for validation.

Previous research has underscored the significance of radiation as a pivotal risk factor for differentiated thyroid cancer (DTC) (20). Notably, a history of radiation exposure to the thyroid during childhood is regarded as the most perilous factor in precipitating DTC (21, 22). Studies indicate that susceptibility to radiation increases with younger age, amplifying the associated risk (22). Hence, what repercussions does radiation exact on intraglandular dissemination? Nikiforov Y et al. led the way in investigating this aspect. They delved into the tumor morphology and clinical features of 84 cases of thyroid cancer, aged 5–14 years, in the aftermath of the Chernobyl disaster. Their findings uncovered a notable trend of heightened invasiveness in these tumors, with an intraglandular dissemination rate soaring to 92%, alongside capsular and adjacent tissue invasion at 89%, and neck lymph node metastasis at 88% (23). These outcomes strongly suggest that radiation exposure not only poses a substantial risk for DTC, but may also serve as a significant predisposing factor for intraglandular dissemination.

In recent years, numerous studies have focused on the correlation between intraglandular dissemination and lymph node metastasis, revealing a closely intertwined relationship. In 2013, Jung YY et al. conducted a univariate analysis of 229 cases of papillary thyroid carcinoma (PTC) patients with lymph node metastasis, unveiling a noteworthy correlation between intraglandular dissemination (P = 0.001) and lymph node metastasis (24). Subsequently, in 2015, Kust D et al. analyzed 97 PTC patients, delineating distinct groups including 64 with nodular goiter (NG), 28 with intraglandular dissemination, 26 with Hashimoto’s thyroiditis (HT), and 7 with follicular or eosinophilic adenomas. Their findings revealed that PTC patients with intraglandular dissemination but devoid of NG or HT were more predisposed to lymph node metastasis (25). Additionally, Lei Y, Yue C, Punda A, Džepina D, and others explored the relationship between intraglandular dissemination and lymph node metastasis across various cohorts, consistently aligning with previous research outcomes (5, 14, 26, 27). In 2021, Qian B et al. conducted an in-depth analysis of the association between intraglandular dissemination and lymph node metastasis. They collected and juxtaposed clinical and pathological data from 117 matched cases with intraglandular dissemination and 204 cases without intraglandular dissemination at Wuhan Union Hospital. The results not only underscored a significantly higher lymph node metastasis rate in PTC patients with intraglandular dissemination compared to those without intraglandular dissemination, but also revealed a substantially greater number of lymph node metastases in the intraglandular dissemination group compared to the non-intraglandular dissemination group (7.92 vs. 4.23). They posited that intraglandular dissemination serves as a risk factor for lymph node metastasis, advocating for a more comprehensive lymph node dissection during surgery for such patients (11).

Different studies have consistently demonstrated a strong correlation between intraglandular dissemination and lymph node metastasis, prompting researchers to explore the feasibility of developing relevant clinical prediction models. Can intraglandular dissemination be utilized as a predictor for lymph node metastasis? Yue C et al., through an analysis of the pathological characteristics of 153 cases of papillary thyroid carcinoma (PTC) at Beijing Tongren Hospital affiliated with Capital Medical University from January 1, 2006, to December 31, 2013, found a correlation between intraglandular dissemination and lymph node metastasis. However, intraglandular dissemination was not identified as an effective predictor for lymph node metastasis (26). Similarly, Punda A et al. demonstrated that intraglandular dissemination is not a statistically significant predictor for lymph node metastasis (27). Although researchers have not succeeded in establishing such a clinical prediction model, the relationship between intraglandular dissemination and lymph nodes cannot be overlooked. Preoperative determination of intraglandular dissemination status holds significant importance for determining the extent of lymph node dissection during surgery.

## 3 The pathological features of thyroid cancer with intraglandular dissemination

What pathological characteristics does intraglandular dissemination exhibit under the microscope compared to typical thyroid cancer? Jin H et al. described the pathological characteristics of intraglandular dissemination in 2018, suggesting that the primary lesion is centrally located, with smaller lesions arranged radially around the primary lesion, and the number of lesions decreases as the distance from the central lesion increases (6). Pancer J et al. also identified similar pathological features, including a centrally located main tumor, numerous small cancer lesions exhibiting radial distribution (with decreasing satellite lesion density as distance from the main lesion increases), and homogeneous BRAF mutations (7).

Knezević-Obad et al. conducted an analysis of the morphological characteristics of 100 cases of classical papillary thyroid carcinoma (PTC) to determine whether specific morphological features are associated with intraglandular dissemination. However, the results showed no significant cytological features that could indicate the occurrence of intraglandular dissemination (28). In addition to its close association with lymph node metastasis, intraglandular dissemination is also closely correlated with other high-invasiveness pathological features. Lei Y et al. analyzed specimens from 73 cases of PTC (25 cases of intraglandular dissemination PTC, 17 cases of multifocal non-intraglandular dissemination PTC, and 31 cases of solitary PTC) obtained through total thyroidectomy. They found that thyroid cancer with intraglandular dissemination was associated with a higher rate of capsule invasion and vascular invasion (5).

While papillary thyroid carcinoma (PTC) generally has a favorable prognosis, some PTC patients present with higher invasiveness and a poorer prognosis. This encompasses high-cellular variants, diffuse sclerosing variants(DSV), columnar cell variants, and PTC with micropapillary structures (29). Do the probabilities of intraglandular dissemination occurrence remain consistent across these subtypes? Existing research suggests that intraglandular dissemination is prevalent in the diffuse sclerosing variant of PTC, implying a close association (4, 30). Gómez-Morales et al. identified, through electron microscopy, extensive lymphocyte infiltration as a hallmark of the diffuse sclerosing variant, this inflammatory infiltration phenotype can trigger a tumor-specific immune response, which in turn can cause an autoimmune response related to non-neoplastic thyroid follicles. And it may be one of the reasons promoting intraglandular dissemination (30). Therefore, extensive lymphocytic infiltration may be a key pathological morphological differentiation point between DSV and intraglandular dissemination, which can be a direction for future study. Additionally, the specific manifestations of intraglandular dissemination in other subtypes of PTC remain ambiguous, so more research may be conducted on this topic.

## 4 The protein expression of thyroid cancer with intraglandular dissemination

Many studies have shown that thyroid cancer with intraglandular dissemination is more prone to lymph node metastasis and is associated with poor prognosis. This has prompted researchers to consider whether there are aberrantly expressed proteins associated with intraglandular dissemination. What are the specific mechanisms involving these proteins? Can they be used for targeted therapy for this type of thyroid cancer in the future? However, there are relatively few reports on the molecular mechanisms of thyroid cancer with intraglandular dissemination currently available.

Scaffold proteins are crucial regulators of signaling networks, and their aberrant expression may lead to dysregulation of signaling pathways, thereby triggering tumor development (31). Among scaffold proteins, immunophilins play a significant role by interacting with proteins and guiding their proper assembly (32, 33). FK506 binding proteins (FKBPs) act as immunophilins and play unique roles in multiple signaling pathways and tumor immune evasion (32, 34). FKBP5 has been shown to play important roles in various tumors such as gastric cancer, renal clear cell carcinoma, and prostate cancer (35–37). Gao et al. conducted immunohistochemical analysis on 115 pairs of papillary thyroid carcinoma (PTC) tissues and normal tissues, revealing elevated expression of FKBP5 in PTC tissues. Further analysis of FKBP5 expression levels and clinical pathological features of PTC revealed a correlation between high FKBP5 expression and increased intraglandular dissemination rate and poor prognosis. Cell experiments demonstrated that high expression of FKBP5 promotes proliferation and inhibits apoptosis of thyroid cancer cells (thyroid cancer cells:TPC-1 and KTC-1 cell lines). Conversely, FKBP5 deficiency yielded opposite results, as knocking out FKBP5 significantly reduced the expression levels of proliferation markers PCNA and Ki67. Furthermore, animal experiments indicated that high expression of FKBP5 promotes tumor growth in mice, while mice with reduced fkbp5 expression showed significantly slowed tumor growth (38).

CENPF is a specific nuclear antigen closely associated with the cell cycle. It is minimally expressed in the G0/G1 phase, accumulates in the nuclear matrix during the S phase, and reaches its peak expression in G2/M phase cells (39). Research has revealed the significant role of CENPF in various tumors. Prior studies have indicated a correlation between CENPF overexpression and bone metastasis in breast cancer (40). Its aberrant expression also plays a crucial role in the occurrence and development of cervical cancer, gastric cancer, liver cancer, and renal cancer (41–44). Han et al. conducted immunohistochemical analysis to detect CENPF expression in papillary thyroid microcarcinoma (PTMC), revealing upregulation of CENPF expression in PTMC. High expression of CENPF has been demonstrated to be associated with T staging and intraglandular dissemination. Analysis of The Cancer Genome Atlas (TCGA) database showed lower overall survival and disease-free survival rates in the group with high CENPF mRNA expression. Furthermore, cell experiments have shown that silencing CENPF can inhibit proliferation, induce cell cycle arrest, and promote apoptosis in thyroid cancer cells (thyroid cancer cells:TPC-1 and KTC-1 cell lines). Animal experiments have also demonstrated that high expression of CENPF can promote tumor growth (45).

CX26 is one of the members of the gap junction formation protein family. Previous studies suggested that it could inhibit tumor growth (46, 47). However, recent research has found that this junctional protein can facilitate intercellular communication, thereby promoting tumor initiation and progression, especially in close association with tumor metastasis (48, 49). McLachlan et al. proposed that junctional proteins exert inhibitory effects on primary tumors in breast cancer, but in advanced tumors, they may also promote tumor development (50). Naoi et al. examined the expression of CX26 in surgical specimens from 69 cases of papillary thyroid carcinoma (PTC), 11 cases of follicular thyroid carcinoma (FTC), and 22 cases of follicular thyroid adenoma (FTA), and investigated the relationship between CX26 expression and its clinical pathological features. The results showed that CX26 expression was negative in FTA and normal thyroid tissue, while it was positive in 47.8% of PTC and 45.5% of FTC cases. The study further indicated that the probability of intraglandular dissemination (30.3% vs. 11.1%) and lymphatic vessel invasion was higher in the CX26-positive PTC group compared to the CX26-negative group. Therefore, the researchers suggested that CX26 may be associated with the pathogenesis of PTC and FTC, and closely related to their invasiveness (51). However, this study has not been further explored, and future validation could be done through cell experiments and animal studies.

Kinesin family member 11 (KIF11) is a molecular motor protein that regulates the formation of the spindle apparatus, thereby modulating the cell cycle progression (52). Han et al. conducted immunohistochemical analysis to assess the expression of KIF11 in thyroid cancer, categorizing it into high-expression and low-expression groups, and analyzed the relationship between KIF11 expression and clinical pathological features. The results indicate that high expression of KIF11 is associated with T staging and intraglandular dissemination. Cell experiments demonstrate that knocking out KIF11 can inhibit cell proliferation (thyroid cancer cells: TPC-1 and KTC-1 cell lines), promote apoptosis, and induce cell cycle arrest. Animal experiments show that the tumor volume significantly decreases in the KIF11 knockout group (53).

Protein tyrosine kinase 7 (PTK7) is a member of the receptor protein tyrosine kinase family, initially identified as an inactive kinase in colon cancer cells and melanoma cells (54). PTK7, as a transmembrane receptor protein, participates in the transmission of signals in the Wnt and VEGF pathways (55). Studies have found that PTK7 is not expressed or is expressed at low levels in most normal tissues, but is highly expressed in tumors such as colon cancer, breast cancer, ovarian cancer, and lung cancer, playing an important role in tumorigenesis and development (56–59). Duan et al. conducted immunohistochemical analysis of PTK7 expression in 79 pairs of thyroid cancer tissues and adjacent tissues. The results showed that PTK7 expression in cancer tissues was significantly higher than in adjacent tissues. The relationship between PTK7 and clinical pathological features showed a significant correlation between PTK7 expression levels and TNM staging and intraglandular dissemination. Additionally, cell experiments indicated that PTK7 can promote cell proliferation (thyroid cancer: TPC-1 and KTC-1 cell lines) and inhibit apoptosis, while animal experiments showed that the tumor volume in the PTK7 knockout group was significantly smaller than that in the control group. Thus, PTK7 is involved in the progression of thyroid cancer and holds promise as a new therapeutic target (60).

Currently, there is relatively little research on the protein aspects of thyroid cancer with intraglandular dissemination, and previous studies have mostly remained at the level of immunohistochemistry, with shallow exploration. It is unclear which signaling pathways specific aberrant proteins are involved in and whether there are interactions between proteins. In fact, emerging methods such as proteomics can be utilized for relevant research in the future, providing possibilities for exploring its mechanisms and targeted therapy.

## 5 The identification of thyroid cancer with intraglandular dissemination

Previous research results indicate that thyroid cancer with intraglandular dissemination has a higher likelihood of lymph node metastasis, higher rates of capsule invasion, higher rates of vascular invasion, and a greater likelihood of BRAF V600E mutation. This suggests that this type of thyroid cancer has a stronger invasive ability, greater metastatic potential, and a poorer prognosis. Therefore, correctly identifying thyroid cancer with intraglandular dissemination is particularly important, especially preoperatively, as it is more conducive to evaluating the surgical approach.

### 5.1 Preoperative identification

Currently, the primary preoperative assessment method for thyroid cancer is neck thyroid + lymph node ultrasound. In fact, as early as 1988, Yokozawa T and others analyzed the correlation between preoperative ultrasound manifestations of thyroid cancer and pathological features of surgical specimens, showing that the results were beneficial for the diagnosis of intraglandular dissemination. Preoperative ultrasound detected intraglandular dissemination in 22 out of 87 patients. The probability of preoperative ultrasound diagnosing intraglandular dissemination was closely related to the size of the lesion (61). It can be seen that preoperative ultrasound is important and superior for the evaluation of thyroid cancer. In addition, researchers have indicated that when preoperative ultrasound fails to aid in diagnosis, methods such as CT and FNA may be helpful. Especially when the tumor diameter is large, even if no multiple lesions are found by ultrasound, the possibility of intraglandular dissemination cannot be ruled out, and CT and FNA can be used as supplementary examinations. In fact, although the preoperative diagnosis of PTC is relatively easy, diagnosing intraglandular dissemination preoperatively is very challenging, even combining the three preoperative examination methods. Therefore, further research is warranted to improve preoperative detection of intraglandular dissemination (6).

### 5.2 Intraoperative identification

When preoperative ultrasound, CT, and FNA do not indicate intraglandular dissemination but cannot exclude it, intraoperative rapid frozen section examination is of significant importance (6). Intraoperatively, performing frozen section examination on the specimen and observing whether a large number of small lesions with radial distribution appear adjacent to the main tumor focus under the microscope are important identification points for intraglandular dissemination (6, 7).

### 5.3 Postoperative identification

While preoperative identification of thyroid cancer with intraglandular dissemination holds significant importance, it’s crucial not to overlook postoperative identification and assessment for cases where successful identification wasn’t achieved preoperatively or intraoperatively. This thorough postoperative evaluation could inspire more tailored and proactive treatment approaches. Researchers have already utilized diverse methods to analyze tumor lesions and judge the clonal origin of multifocal thyroid cancer.

BRAF V600E is the most common mutation in thyroid cancer, occurring in approximately 60% of papillary thyroid carcinomas (PTC) (62). Previous studies have shown that BRAF V600E is associated with adverse factors such as lymph node metastasis and local recurrence (63). Researchers like Lei Y have found a correlation between intraglandular dissemination and BRAF V600E mutation rates (5). Similarly, studies by Tallini G have confirmed through both univariate and multivariate analysis that BRAF V600E mutation is closely related to intraglandular dissemination (64). Many researchers have therefore used the status of BRAF V600E in tumor lesions to determine whether intraglandular dissemination has occurred. In 2010, Wang W et al. evaluated the clonal origin of 25 pairs of bilateral thyroid cancers (synchronous or metachronous) and 15 pairs of matched metastatic lymph nodes (using BRAF gene mutation analysis and X chromosome inactivation detection). The BRAF mutation status was consistent in 18 out of 21 thyroid tumors (positive in 12 cases, negative in 6 cases), and in 12 out of 15 patients, the BRAF status of metastatic lymph nodes matched that of their primary tumors. Eleven patients were suitable for X chromosome inactivation assay, and the results showed that 9 out of 11 patients exhibited the same inactivation pattern in bilateral tumors. This data suggests that bilateral, recurrent, and metastatic papillary thyroid carcinomas usually originate from intraglandular dissemination of a single tumor (65). In 2018, Jovanovic L et al. assessed 55 tumor lesions from 18 cases of multifocal PTC for genome-wide allelic imbalances (AI) and BRAF V600E mutation status. The results showed that genetic changes were consistent with monoclonality in 15 out of 18 (83%) cases, indicating that these 15 cases were also caused by intraglandular dissemination. Although most of these cases eventually exhibited morphologically diverse tumor lesions, phylogenetic analysis indicated that this morphological diversity was the result of subclonal progression (66). It is evident that multifocal thyroid cancers with morphological diversity may also be caused by intraglandular dissemination. Although BRAF mutation is the most common mutation in thyroid cancer, analyzing only the BRAF mutation status to determine tumor origin may lead to an increased positivity rate for intraglandular dissemination. In order to make the research results more accurate, Bansal M conducted a comprehensive analysis of point mutations in BRAF, NRAS, HRAS, KRAS, and rearrangements in RET/PTC1 and RET/PTC3 (in 60 cases of multifocal PTC), revealing that 25% of cases had all lesions with the same mutation, suggesting that these 25% of cases may be caused by intraglandular dissemination. Additionally, histopathologically, researchers found that these lesions were often located within the same thyroid lobe (67). In addition to the aforementioned methods, the latest whole-exome gene sequencing methods have also been used for evaluation. Lu Z et al. assessed the tumor origin of 8 cases of multifocal papillary thyroid cancer (MPTC) using whole-exome gene sequencing and found that 25% of MPTC cases may have a monoclonal origin. However, the tumor lesions of the MPTC patients in this study were relatively large, especially in the 2 cases of MPTC that showed monoclonal origin, with tumor diameters reaching up to 1 cm. This does not align with the pathological characteristic of intraglandular spread proposed in previous studies (68). Therefore, it cannot be concluded that 25% of MPTC cases are caused by intraglandular dissemination. Based on these various methods, it can indeed provide significant help in identifying intraglandular dissemination, but researchers must avoid significant bias when selecting patients to be included in the study.

## 6 The clinical significance of correct identification

Preoperative identification of intraglandular tumor dissemination, achieved through auxilliary methods like ultrasound and CT scans, holds significant importance in determining the surgical approach.In such cases, clinicians should consider total thyroidectomy for patients (69). This not only reduces the likelihood of secondary surgery, but also enables adjunctive I131 therapy for some patients based on routine pathological results. Considering intraglandular dissemination as a risk factor for lymph node metastasis, a more thorough lymph node dissection should be performed during surgery.

Next, when intraglandular tumor dissemination is confirmed through rapid frozen section analysis during surgery, it prompts a reconsideration of the surgical approach. If a patient had initially opted for “partial thyroidectomy” before the operation, clinicians should engage in comprehensive communication with the patient’s family during the procedure. They should explain to the patient’s family that “total thyroidectomy” would be more suitable and seek their consent for a potential modification in the surgical approach, involving total thyroidectomy and a more extensive cervical lymph node dissection.

When intraglandular dissemination is confirmed through routine pathology, genetic sequencing, or other methods postoperatively, it remains of significant importance. For patients who have undergone total thyroidectomy, clinicians may consider I131 therapy based on the specific circumstances. For patients who have undergone partial thyroidectomy, clinicians should assess postoperative conditions to consider the necessity of a secondary surgery. Alternatively, they can follow patients more closely, if abnormalities are found during the follow-up, surgery can be performed again.

Although the current risk stratification for thyroid cancer does not include intraglandular dissemination as a factor, and research on the correlation between intraglandular spread and I131 is also lacking, previous studies have shown a close association between intraglandular dissemination and lymph node metastasis, extrathyroidal invasion, and BRAF V600E mutation (5, 64). Therefore, clinicians should carefully consider the impact of intraglandular dissemination when assessing the need for postoperative I131 therapy.

## 7 Conclusion

In summary, thyroid cancer with intraglandular dissemination is more common in young patients and is associated with high rates of lymph node metastasis, capsular invasion, vascular invasion, and BRAFV600E mutation. Although some proteins have been found to be aberrantly expressed in this condition, the underlying mechanisms remain unclear, necessitating further research for elucidation. Moreover, the accurate identification of this condition is crucial, and intraglandular dissemination should be regarded as an extremely invasive biological behavior. Patients with this condition require more proactive treatment and closer follow-up.

## Author contributions

YZ: Supervision, Writing – original draft, Writing – review & editing. YD: Supervision, Writing – review & editing. MZ: Supervision, Writing – review & editing. BW: Conceptualization, Supervision, Writing – review & editing. JZ: Conceptualization, Supervision, Writing – review & editing.
